# Evaluation of the serum level of estrogen, progesterone, prolactin, and testosterone in patients with trigeminal neuralgia compared to a healthy population

**DOI:** 10.1002/cre2.815

**Published:** 2023-11-28

**Authors:** Fatemeh Lavaee, Sahar Didar, Aylar Afshari

**Affiliations:** ^1^ Oral and Maxillofacial Disease Department, Oral and Dental Disease Research Center, School of Dentistry Shiraz University of Medical Sciences Shiraz Iran; ^2^ Student Research Committee, School of Dentistry Shiraz University of Medical Sciences Shiraz Iran

**Keywords:** hormone, nerve conduction, neuralgia

## Abstract

**Objectives:**

The goal of this study is to measure and compare the hormonal serum levels (estrogen, progesterone, testosterone, prolactin, dihydrotestosterone [DHT]) in trigeminal neuroglia (TN) menopausal women and healthy women.

**Materials and Methods:**

This cross‐sectional and case‐control study was performed in 2018 and 2019. For this study, menopausal women with confirmed TN were enrolled. Twenty‐two healthy women in the control group and 19 in the case group participated. Blood samples were taken from participants for assessment of hormonal serum levels (estrogen, progesterone, testosterone, prolactin, DHT). Data were analyzed by SPSS version 18. Mann−Whitney, *T*‐test, kormography test, nonmetric, *χ* ^2^ test, and odds ratios have been used.

**Results:**

In patients with TN, the serum level of testosterone was significantly higher (*p* = .036), and the serum level of prolactin (*p* = .016) was significantly lower. Other evaluated hormones' serum level was identical in the two groups. Patients with abnormal estrogen levels were more in the TN group in comparison with the healthy group. The abnormality of progesterone in TN patients was more in comparison to the healthy control group.

**Conclusions:**

Estrogen and progesterone serum levels in TN patients are higher in comparison with the healthy group, while prolactin and testosterone serum levels are lower in the control group. Moreover, the DHE serum level is similar in both groups.

## 1 INTRODUCTION

Trigeminal neuralgia (TN) is a neuropathic pain that causes sudden, brief, stabbing, and recurrent pain in local and small parts of the face (Amanat et al., 2013; Derafshi et al., 2019; Zakrzewska, 2010). TN usually begins in the middle or old age range. Although, it may occur in young adults and children as well. The attacks can occur repeatedly and last for a few seconds. The attacks may begin when the trigger zone is stimulated, which can be placed within the trigeminal nerve pathway (Lavaee et al., 2021; Love & Brain, 2001). TN has different etiologies, causing demyelination in the trigeminal zone. Neurovascular compression, multiple sclerosis, tumors, cysts, and diabetes mellitus are the most common causes of this disorder (International KT‐OS, 2007 U, 1963; Lavaee et al., 2022). In some studies, this disease's neuropathic and neurotropic role has been related to sex hormones. The effect of these hormones on the quality and rate of nerve conduction has been reported as well. These studies have shown a higher incidence of peripheral neuropathy in menopausal females (Warren et al., 2015). Peripheral sensory and autonomic neurons express estrogen receptors (Jeon, 1961). The nerve transmission speed depends on velocity and latency (the duration between applying stimulation and the record of wave formation in nerve conduction studies). On the other hand, studies showed that the degree of myelination can significantly affect velocity and latency in the nerve conduction (Takeo & Sakuma, 1995). In a study, sex hormone replacement therapy resulted in faster velocity and shorter latency, which can be indicative of a possible association between sex hormones and nerve myelination (Kim et al., 2011). Another study investigated whether estrogen (E2) shows a favorable recovery effect on nerve injury. This study indicated that mice with induced nerve injury treated by local injection of E2 had greater nerve conduction velocity and vascularity than the placebo group (Sekiguchi et al., 2012). Singh et al. (2016) explored the relationship between the serum level of estrogen and progesterone and peripheral motor nerve neuropathy by motor nerve conduction velocity (MNCV) in postmenopausal women. Their findings reported a lower level of serum estrogen in postmenopausal women with peripheral neuropathy.

According to the information mentioned above and the higher prevalence of some neuropathic pains, such as burning mouth syndrome and TN in females with more variant sex hormone status, it is important to evaluate the hypothesis of a possible relationship between sex hormones and the prevalence of TN. To the best of our knowledge, there is no study on sex hormones in patients with TN. Therefore, in this study, the serum levels of FSH, LH, testosterone, estrogen, and progesterone in females affected by TN who had been referred to the Oral and Maxillofacial Disease Department of Shiraz Dental School were evaluated.

## 2 MATERIALS AND METHODS

This cross‐sectional case‐control study was done in 2018 and 2019. The protocol of this study was approved by the ethics committee of Shiraz University of Medical Sciences (IR.SUMS.REC.1397.993). For the study, menopausal women with confirmed TN who had been referred to Emam Reza clinic and Oral and Maxillofacial Disease Department of Shiraz Dental Faculty were enrolled. A written consent form was obtained from each participant. Participants who had any disease or strange nutritional habits and environmental factors affecting the serum level of sex hormones were excluded from the study. The blood samples were obtained by an expert nurse. The day of blood sampling for menopausal women was not specific. The participants had to consider 2 h between waking up and blood samples obtaining. The serum levels of dihydrotestosterone (DHT), prolactin, testosterone, progesterone, and estrogen were evaluated. Patients' demographic data including, age, other systemic diseases, and menopausal situations, were registered. Nineteen women with TN were recruited in the case group, and 21 healthy women with the same age, environmental, and nutritional status as the case group were enrolled in the control group. The participants in the control group were patients who had been referred to Shiraz Dental School for routine dental evaluation.

The mean hormone serum level was calculated and then compared between the two groups. Measured hormones were estradiol, progesterone, testosterone, prolactin, and dihydrotestosterone.

Data were analyzed in SPSS version 18. This study used the kymograph test for assessing the normality of data distribution. Nonparametric test of the Mann−Whitney test was used for comparing the mean of evaluated hormones. Moreover, the Student *t*‐test was used for comparing the mean age of two groups and the *χ* ^2^ test for comparing the abnormality rate of hormonal ranges. The odds ratio was reported for the risk estimation affected by TN.

## 3 RESULTS

In this case‐selection study, 41 menopausal women were enrolled in two groups (healthy control [22 participants] and TN [19 participants]). The age of both groups was matched with each other (*p* = .72). The mean age in the control group was 54.09 ± 7.009 and 55.00 ± 8.75 in the TN group.

The data on hormonal levels did not have a normal distribution due to the Kolmogorov−Smirnov test. The serum level of each evaluated hormone and its comparison between the two groups is shown in Table 1. While the serum level of testosterone was significantly higher (*p* = .036) in patients with TN, the serum level of prolactin (*p* = .016) was significantly lower. The serum levels of other evaluated hormones were identical in the two groups.

**Table 1:** The serum level of each evaluated hormone and its comparison between the two groups.

Groups		ESTRO^a^ (ng/mL)	PROGES^b^ (ng/mL)	TESTOS^c^ (ng/mL)	PRL (ng/mL)^d^	DHT (pg/mL)
Control						
	*N*	22	22	22	22	22
	Mean	49.81	0.41	0.24	16.46	217.00
	Standard deviation	18.54	0.19	0.22	7.34	125.75
	Median	47.85	0.39	0.18	15.30	177.50
TN						
	*N*	19	19	19	19	12
	Mean	46.55	1.29	0.38	11.00	233.63
	Standard deviation	50.79	2.26	0.19	3.83	131.24
	Median	32.90	0.57	0.33	11.41	208.80
	*p* Value	.13	.10	.01	.01	.77

Abbreviations: DHT, dihydrotestosterone; TN, trigeminal neuroglia.

^a^ESTRO, estrogen; ^b^PROGES, progesterone; ^c^TESTOS, testosterone; ^d^PRL, prolactin.

The normality assessment of the serum level of each hormone and its comparison between the two groups are presented in Table 2. While estrogen and progesterone serum levels were significantly abnormal in TN patients, prolactin and testosterone were significantly abnormal in healthy control (*p* < .05). Abnormal DHT serum levels in both evaluated groups were not statistically different (*p* < .05).

**Table 2:** Normality assessment of the serum level of each hormone and its comparison between the two group.

Groups	ESTRO^a^	PROGES^b^	TESTOS^c^	PRL^d^	DHT
	Abnormal	Abnormal	Abnormal	Abnormal	Abnormal
Case	31.8%	0%	77.3%	31.8%	45.5
	7	0	17	7	10
TN	68.4%	21.1%	42.1%	0%	58.3%
	13	4	8	0	7
*p* Value	.02	.03	.02	.03	.36

Abbreviations: DHT, dihydrotestosterone; TN, trigeminal neuroglia.

^a^ESTRO, estrogen; ^b^PROGES, progesterone; ^c^TESTOS, testosterone; ^d^PRL, prolactin.

## 4 DISCUSSION

Evaluation of patients with TN showed that the serum level of TN women in comparison with healthy participants was significantly higher. In addition, estrogen and progesterone serum level abnormality were more common in patients with TN than in healthy ones. In contrast, the serum level abnormality of prolactin, and testosterone was more in the control group.

The effect of sex hormones on neurons has been evaluated in previous studies in the literature. Their reported findings were controversial. Some studies indicated a neuroprotective role for these hormones (Suzuki et al., 2006), while others did not confirm this role (Sekiguchi et al., 2012, 2013; Wise et al., 2001).

In a study, the relationship between the status of the motor nerve (peripherally) and serum levels of estrogen and progesterone in postmenopausal females was evaluated by MNCV. Although it was observed that the serum level of estrogen in postmenopausal patients with peripheral neuropathy in comparison with the healthy group was significantly lower, no critical effect on progesterone was reported by (Solmaz et al., 2017). In another study, higher levels of progesterone were accompanied by decreased optic nerve conduction velocity (Gundogan et al., 2013). Also, in an evaluation, an elevated level of progesterone was considered to be effective in decreasing the nerve conduction velocity (Amir & Fessler, 2013; Henderson & Popat, 2011). On the other hand, the animal model evaluation did not indicate any noticeable influence of estrogen and progesterone therapy on nerve repair (Nachemson et al., 1985).

The findings of these studies are in accordance with what we reported in the current study. In menopausal participants who are considerably more prone to neuropathies, no significant difference was reported for estrogen and progesterone between the patients with TN and the healthy control group; although, these hormones were more distributed in an abnormal range in TN patients.

On the other hand, some other studies showed neuroprotective and neurotrophic properties for progesterone. This role was reported in electrophysiological alteration of diabetic‐induced neuropathy in rats (Hale & Burger, 2009; Kim et al., 2011). In several studies, this neuroprotective effect is also reported for estrogen. A study proposed the protective effect of estrogen against neural death mediated by estrogen receptors (Leonelli et al., 2007; Yin et al., 1998).

It is suggested that estrogen can also regenerate the damaged nerves or enhance nerve velocity and vascularity. In the English literature, several mechanisms for neuroprotectivity have been proposed. For example, progesterone can induce regeneration and nerve remyelination, which can have a significant part in the pathogenesis of most neuropathies (Chan et al., 1998; Keirstead et al., 2005; Koenig et al., 1995). In damaged nerves, progesterone prevents secondary neural loss by decreasing edema, inflammatory cytokines, and reactive gliosis (Chan et al., 2000; Groyer et al., 2006; Jung‐Testas et al., 1996; Magnaghi et al. 2022; Sereda et al., 2003). Several studies reported that estrogen treatment could increase vascular epithelial growth factor expression, which suggested the proangiogenic properties of estradiol (Lubetzki et al., 1993). The results of the present study, rather than confirming the neuroprotective role of progesterone and estrogen, showed an imbalance of sex hormones in patients with TN.

Prolactin excretes from the anterior pituitary, neuronal and non‐neuronal cells of the peripheral nervous system and central nervous system (Baumann & Pham‐Dinh, 2001). Prolactin receptors (long isoform) are absent in trigeminal afferent nerves of the dental pulp tissue, while they can be detected in the glial cells of the myelin sheath (Ghoumari et al., 2002). Prolactin induces action potential firing. It can control neuronal excitability and circuitries by its rapid neuronal responses (Dusart et al., 1997). In our study, the number of patients with an abnormal rate of prolactin was less in the TN group than in the control group. However, no significant differences were observed between TN patients and healthy ones.

Testosterone can induce nerve regeneration and neuronal formation. It can also accelerate axon outgrowth, which may lead to nerve regeneration. Some molecules involved in steroidogenesis, such as translocator protein‐18KDa (TSPO), may participate in neuropathic pain control. These neuropathic steroids have shown antinociceptive effects in animal models (Ghoumari et al., 2002). In an evaluation, testosterone propionate administration increased the number of motoneurons in the hypoglossal nucleus in the postoperative phase (Notterpek et al., 1993). Moreover, its regenerative effect on neurons has been reported. It seems that the results of the present study do not follow the mentioned effect of testosterone on neuropathic pain since the serum level of this hormone was more in patients struggling with TN than in the healthy group. Also, the abnormality rate was lower in patients with TN.

DHT is a metabolite of testosterone; it was reduced in diabetic rats in an animal model study. This study proposed that DHT affects the mechanical threshold of nociceptors and induces an analgesic effect in the threshold of nociceptors (Baulieu & Schumacher, 1997; Hamada et al., 2006; Roof et al. 1993, 1994; VanLandingham et al., 2006). In our study, the number of patients with an abnormal rate of DHT was less in the TN group than in the control group. However, no significant difference was observed between the TN patients and healthy ones.

Sex hormonal profile evaluation in patients affected by TN and improving these imbalances may affect the patient's response to routine treatment.

The sample size of our study was small due to financial limitations and the low prevalence of TN; therefore, it is wise to recruit a larger sample size. To the best of our knowledge, there is no similar study on TN patients. Previous studies evaluated different types of neuropathies by several methods and designs; also, they didn't consider sex differences in sex hormone serum levels. These diversities of the methodological structure of studies make the comparison difficult and even not precise. Having different sexes in a study makes comparison impossible. This can explain the controversies of the findings and the indication of sex hormonal imbalances in these patients. Perhaps the small sample size in our study limited our study in discriminating the quality of these imbalances and their possible association with the pathogenesis of TN.

Further case‐control studies with larger sample sizes and recruiting men are suggested for future studies. Moreover, it is suggested to evaluate and analyze all the factors that are effective to the result such as height and weight of the patients (because BMI is effective to sex hormones level), and environmental factors. Furthermore, sexual steroid hormones may have a role in obesity and disorders associated with metabolism.

## 5 CONCLUSION

While testosterone has been increased significantly in patients with TN, prolactin has been decreased. Moreover, progesterone and estrogen were abnormal in patients with TN which confirms the sex hormones imbalance in TN patients.

## AUTHOR CONTRIBUTIONS

**Fatemeh Lavaee**: Study design, patient selection, data gathering, data analysis, writing the draft, and critical editing. **Sahar Didar**: Study design, patient selection, data gathering, data analysis, and writing the draft. **Aylar Afshari**: Data gathering, data analysis, and critical editing.

## CONFLICT OF INTEREST STATEMENT

The authors declare no conflict of interest.

## Data Availability

The authors have nothing to report.
